# Biliary Obstruction Secondary to Transjugular Intrahepatic Portosystemic Shunt Presenting as Alcohol‐Associated Hepatitis

**DOI:** 10.1155/crgm/6660623

**Published:** 2026-03-15

**Authors:** Yash Hegde, Dana Ley, Adnan Said, Prasad Dalvie, Mark Benson, Deepak V. Gopal

**Affiliations:** ^1^ Department of Medicine, School of Medicine and Public Health, University of Wisconsin—Madison, Madison, Wisconsin, USA, wisc.edu; ^2^ Division of Gastroenterology and Hepatology, Department of Medicine, School of Medicine & Public Health, University of Wisconsin—Madison, Madison, Wisconsin, USA, wisc.edu; ^3^ Department of Radiology, School of Medicine & Public Health, University of Wisconsin—Madison, Madison, Wisconsin, USA, wisc.edu

**Keywords:** alcohol-associated hepatitis, biliary obstruction, transjugular intrahepatic portosystemic shunt

## Abstract

Transjugular portosystemic intrahepatic shunt (TIPS) is a procedure used to alleviate portal hypertension. The two most common clinical indications for TIPS are secondary prevention of variceal hemorrhage and refractory ascites. Biliary complications, of which biliary‐shunt fistulas are the most common, are very rare. There are very limited data on TIPS placement leading to biliary obstruction. We describe a patient who underwent TIPS placement leading to biliary obstruction, initially thought to have alcohol‐associated hepatitis, who was successfully treated with interventional radiology‐guided percutaneous biliary drain placement.

## 1. Introduction

Transjugular portosystemic intrahepatic shunt (TIPS) is a procedure used to decrease portal hypertension by creating a shunt between a portal vein and hepatic vein for portal decompression [1]. The two most common clinical indications for TIPS placement are refractory ascites and variceal hemorrhage. Other common indications include recurrent symptomatic hepatic hydrothorax, transfusion‐dependent portal hypertensive gastropathy, Budd–Chiari syndrome, and hepatorenal syndrome [2]. Despite TIPS procedures having a high success rate, complications can occur which include shunt thrombosis, stent migration, bleeding, ischemic hepatitis, infection, hepatic encephalopathy, and biliary complications [3]. Clinically significant biliary complications from TIPS placement are exceedingly rare with the most common being biliary‐shunt fistula formations [3]. The literature on biliary obstruction secondary to TIPS placement is very limited with few cases reported. We describe a patient that underwent TIPS placement which was complicated by biliary obstruction and acute liver failure, originally thought to be from alcohol‐associated hepatitis.

## 2. Case Presentation

**FIGURE 1:**
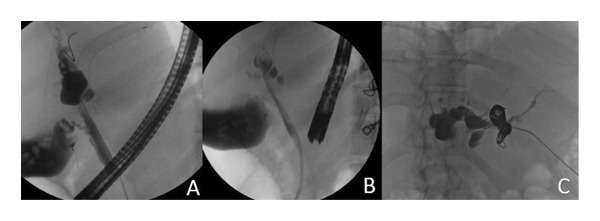
Endoscopic retrograde cholangiopancreatography showing common hepatic duct obstruction secondary to transhepatic intrahepatic portosystemic shunt stent with intrahepatic duct dilation (A) and subsequent stent placement (B). As liver function tests did not improve after stent placement, percutaneous transhepatic cholangiogram with biliary drain was performed (C).

A 52‐year‐old female with alcohol use disorder and decompensated alcohol‐related cirrhosis, with previous hospitalizations for refractory ascites and recurrent episodes of variceal hemorrhage despite variceal band ligation, presented with hematemesis. She was vitally stable. Initial laboratory workup was remarkable for hypoalbuminemia at 2.7 g/dL, elevated alkaline phosphatase at 159 U/L, moderate elevation in aspartate transaminase (AST) at 46 U/L, and normal alanine transaminase (ALT) level at 27 U/L, total hyperbilirubinemia at 1.5 mg/dL, decreased hemoglobin at 8.7 g/dL, and increased international normalized ratio (INR) of 1.9 (Table 1). Her blood alcohol level was 300 mg/dL. Esophagogastroduodenoscopy (EGD) revealed small (< 5 mm) esophageal varices with no stigmata of recent bleeding and multiple gastroesophageal varices due to portal hypertension that were not actively bleeding. She then underwent a TIPS procedure (Viatorr‐covered TIPS device placed into the middle hepatic vein to the left portal vein near the bifurcation, 7 + 2 cm × 8–10 mm covered length) with coil embolization of gastric varices and improvement in her hepatic venous pressure gradient from 15 to 8 mmHg. After TIPS placement, her Model for End‐Stage Liver Disease with sodium (MELD‐Na) score increased and was initially attributed to alcohol‐associated hepatitis given ongoing heavy alcohol use and an elevated blood alcohol level on admission. She was treated with prednisolone 40 mg daily and intravenous N‐acetylcysteine (NAC) for 5 days, but her MELD‐Na score continued to increase. Initial evaluation did not demonstrate biliary obstruction, and ongoing liver function deterioration despite medical therapy prompted repeat cross‐sectional imaging. Abdominal computed tomography (CT) scan showed severe intrahepatic biliary duct dilation secondary to TIPS compression of the central common hepatic duct. She then underwent endoscopic retrograde cholangiopancreatography (ERCP) with placement of a plastic stent (8.5 French by 12 cm) into the common bile duct for decompression (Figure 1). Repeat abdominal CT scan showed improved right‐sided biliary duct dilation with continued severe left biliary duct dilation. Due to her MELD‐Na score continuing to increase after ERCP, interventional radiology placed a left percutaneous transhepatic cholangiography (PTC) drain as a bridge to liver transplant evaluation. Her MELD‐Na score significantly improved after left PTC drain placement, and she was discharged from the hospital with scheduled outpatient liver transplant evaluation. She was ultimately deemed not to be a candidate for liver transplantation and passed away.

**TABLE 1:** Clinical course and laboratory trends following TIPS placement.

Hospital day	Key intervention	MELD‐Na score	Total bilirubin (mg/dL)	Alkaline phosphatase (U/L)	AST (U/L)	ALT (U/L)
0	Pre‐TIPS	21	1.5	159	46	27
5	Post‐TIPS	27	10.2	112	81	36
7	Prednisolone + NAC therapies	29	16.1	138	154	62
13	ERCP + stent	33	27.9	202	105	73
15	PTC drain	30	26.1	216	70	55

*Note:* MELD‐Na, Model for End Stage Liver Disease + Sodium; AST, aspartate aminotransferase, ALT, alanine aminotransferase; NAC, N‐acetylcysteine; ERCP, endoscopic retrograde cholangiopancreatography.

Abbreviations: PTC, percutaneous transhepatic cholangiography; TIPS, transjugular intrahepatic portosystemic shunt.

## 3. Discussion

Clinically severe biliary complications after TIPS placement are rarely documented and occur in less than 1% of cases [3, 4]. The most commonly cited biliary complication after TIPS placement in the literature is the formation of biliary‐shunt fistulas which may result in hemobilia, cholangitis, stent infection, and sepsis [5]. To the best of our knowledge, this is the first reported case of biliary obstruction from a TIPS procedure that clinically mimicked alcohol‐associated hepatitis.

Although alcohol‐associated hepatitis was initially suspected, the patient did not improve with corticosteroid and NAC therapy, and repeat imaging demonstrated biliary ductal dilation from mechanical compression by TIPS. These findings supported biliary obstruction rather than alcoholic hepatitis as the primary cause of liver failure.

The literature on biliary obstruction from TIPS placement is limited. Korrapati et al. presented a patient with Budd–Chiari syndrome who underwent TIPS placement complicated by biliary obstruction [6]. The patient was effectively managed with ERCP and plastic stent placement. In a similar case report, Paterno et al. described a case of a patient with Hepatitis C cirrhosis and portal hypertension who underwent a TIPS procedure that led to obstruction of the common hepatic duct. The patient was treated with PTC with placement of bilateral biliary drainage catheters until liver transplantation [7]. Our case is unique because the patient’s initial presentation with worsening liver enzymes after TIPS placement was thought to be in the setting of alcohol‐associated hepatitis. The patient had ongoing alcohol use as demonstrated by an elevated blood alcohol level on admission. Her liver function tests worsened in the setting of steroid and NAC therapies which prompted repeat abdominal CT imaging revealing biliary obstruction from the TIPS.

PTC and ERCP are integral in the management of patients with biliary complications after TIPS placement. Of these complications, biliary‐shunt fistulas remain the most commonly reported and are managed by using temporary stents to reline hepatic parenchymal tracts, leading to resolution of biliovenous fistulas [8]. In the few case reports describing biliary obstruction from TIPS placement, both stent placement with ERCP and placement of biliary drain catheters through PTC remain as possible solutions for biliary decompression [6, 7]. In our case, adequate biliary decompression was not achieved through ERCP with stenting alone, and left PTC drain placement was required in addition.

In conclusion, we report an unusual case of a TIPS placement complicated by biliary obstruction, which clinically appeared consistent with alcohol‐associated hepatitis. While few previously documented cases of TIPS causing biliary obstruction were treated with ERCP and plastic stenting, percutaneous biliary drainage can additionally be used for ERCP‐refractory cases. Although the patient in our case was at risk of having alcohol‐associated hepatitis given her ongoing heavy alcohol use, it is important to consider TIPS complications as a potential cause for worsening jaundice and liver failure, as the treatment is completely different.

## Author Contributions

Yash Hegde: drafting of case report and critical revision of case report. Dana Ley: critical revision of case report. Adnan Said.: critical revision of case report. Prasad Dalvie: critical revision of case report. Mark Benson: critical revision of case report. Deepak V. Gopal: drafting of case report and critical revision of case report.

Article Guarantor: Deepak V. Gopal, MD, FRCP(C), FACP, FACG, AGAF, FASGE.

## Funding

The authors have nothing to report.

## Disclosure

The case was presented as a poster presentation at the American College of Gastroenterology Annual Scientific Meeting 2024 and won an “ACG Outstanding Poster Presenter” award.

## Consent

Informed patient consent was obtained for publication of case details.

## Conflicts of Interest

The authors declare no conflicts of interest.

## Data Availability

The data that support the findings of this study are available on request from the corresponding author. The data are not publicly available due to privacy or ethical restrictions.
